# Effect of hearing aids on cognitive functions in middle-aged and older adults with hearing loss: A systematic review and meta-analysis

**DOI:** 10.3389/fnagi.2022.1017882

**Published:** 2022-11-14

**Authors:** Zhizhong Yang, Jingnian Ni, Yuou Teng, Mingwan Su, Mingqing Wei, Ting Li, Dongsheng Fan, Tao Lu, Hengge Xie, Wei Zhang, Jing Shi, Jinzhou Tian

**Affiliations:** ^1^Dongzhimen Hospital, Beijing University of Chinese Medicine, Beijing, China; ^2^Department of Neurology, Dongzhimen Hospital, Beijing University of Chinese Medicine, Beijing, China; ^3^Department of Neurology, Peking University Third Hospital, Beijing, China; ^4^School of Life Sciences, Beijing University of Chinese Medicine, Beijing, China; ^5^Department of Neurology, Chinese PLA General Hospital, Beijing, China; ^6^Department of Neurology, Beijing Tiantan Hospital, Capital Medical University, Beijing, China

**Keywords:** hearing aids, hearing loss, cognitive function, Alzheimer's disease, depression

## Abstract

**Objective:**

The study aimed to examine the effects of hearing aids on cognitive function in middle-aged and older adults with hearing loss.

**Data sources and study selection:**

PubMed, Cochrane Library, and Embase were searched for studies published before 30 March 2022. Randomized controlled trials (RCTs) and non-randomized studies of interventions (NRSIs) were included in the search. Restriction was set on neither types, severity, or the time of onset of hearing impairment nor cognitive or psychiatric statuses.

**Data extraction and synthesis:**

Two independent reviewers extracted data and assessed the study quality of RCTs. Cognitive function outcomes were descriptively summarized and converted to standardized mean difference (SMD) in the meta-analysis. Meta-analysis was conducted in RCTs. Sub-group analyses were conducted by cognitive statuses, psychiatric disorders, and cognitive domains.

**Results:**

A total of 15 studies met the inclusion criteria, including five RCTs (*n* = 339) and 10 NRSIs (*n* = 507). Groups were classified as subjects without dementia or with normal global cognition, subjects with AD or dementia, and subjects with depressive symptoms. For subjects without dementia, improvements were found in global cognition, executive function, and episodic memory. For subjects with depressive symptoms, improvements were found in immediate memory, global cognition, and executive function. No improvement was found in subjects with AD or dementia. In total, four RCTs were included in the meta-analysis. For subjects without dementia (SMD = 0.11, 95% confidence interval [CI]: −0.15–0.37) and those with AD, no significant effect was found (SMD = −0.19, 95% CI: −0.65–0.28). For subjects without dementia, no significant effect was found in language (SMD = 0.14, 95% CI: −0.30–0.59) or general executive function (SMD = −0.04, 95% CI: −0.46–0.38). Further sub-group analysis found no significant effect in executive function (SMD = −0.27, 95% CI: −0.72–0.18) or processing speed (SMD = −0.02, 95% CI: −0.49–0.44).

**Conclusion:**

Hearing aids might improve cognitive performance in domains such as executive function in subjects without dementia. The effects on subjects with depressive symptoms remained unclear. No improvement was found in subjects with AD or dementia. Long-term RCTs and well-matched comparison-group studies with large sample sizes are warranted.

**Systematic review registration:**

https://www.crd.york.ac.uk/PROSPERO/, identifier: CRD42022349057.

## Introduction

Nowadays, the rate of hearing loss presents an increasing trend. For example, in China, according to the research by Hu et al. (2022), the prevalence of hearing loss is 50.92%, which is far > 14.3% in the first decade of the 21st century (Fei et al., 2013). Meanwhile, the dementia rate is also increasing among people aged ≥ 65 years (Hu et al., 2022). Hearing loss has been previously regarded as responsible for contributing to symptoms of dementia and cognitive dysfunction in older adults (Uhlmann et al., 1986, 1989). Currently, it has been recognized as a risk factor for dementia (Livingston et al., 2017). In addition, central age-related hearing loss has been thought to be independently related to cognitive frailty, referring to a functional decline coexisting with physical frailty and mild cognitive impairment, thus resulting in an accelerated cognitive decline, increased incident dementia, and other adverse outcomes (Sardone et al., 2021). Hence, hearing treatment and cognitive impairment have received much attention, especially in the aging society (Davies et al., 2017). However, is hearing loss a modifiable risk factor and a possible therapeutic target to improve cognitive function or impede cognitive decline?

Although the mechanism underlying hearing loss and cognitive decline is not yet clear, indeed, studies suggesting improvements in cognition with hearing loss management have been conducted, possibly through enhancing communication and improving the quality of life (Mamo et al., 2018), to prevent isolation and thus to reduce the risk of cognitive decline (Maharani et al., 2019; Griffin et al., 2020). In addition, a long-term cohort study has also indicated that subjects with hearing loss using hearing aids had no difference in cognitive decline compared with people with normal hearing, while those who did not use hearing aids were observed with more severe cognitive decline (Amieva et al., 2015). Nevertheless, there is no recommendation based on high-level evidence yet, leaving major disagreement on the effects of hearing treatment on cognitive decline. Therefore, we conducted a systematic review and meta-analysis to investigate and quantify the effects of a hearing aid—a hearing device that is widely accepted among the population with hearing loss—on middle-aged and older adults with hearing loss with different cognitive statuses and psychiatric disorders to find relatively definite results in certain groups of the population.

## Materials and methods

The systematic review and meta-analysis were conducted according to the Preferred Reporting Items for Systematic Reviews and Meta-Analyses (PRISMA) guidelines (Moher et al., 2009). Our protocol was registered on the PROSPERO International Prospective Register of Systematic Reviews (CRD42022349057).

### Search strategy and selection criteria

Studies published before 30 March 2022 were searched in three electronic databases by two authors (Yang Z and Su M): (1) PubMed, (2) Cochrane Library, and (3) Embase. Keywords included hearing aids, Alzheimer's disease, dementia, mild cognitive impairment, and cognitive function. Details of the search strategy and results are given in Supplementary Table S1.

The inclusion criteria for the articles are as follows: (1) prospective, original articles of randomized controlled trials (RCTs) and non-randomized studies of interventions (NRSIs), which included non-randomized controlled trials (non-RCTs) and pretest–posttest studies using a within-subject design (without control or comparison groups); (2) full-text articles published in English only; (3) articles including subjects aged ≥ 45 years with hearing impairment; (4) those with no restriction on the assessment of hearing status; (5) those with full inclusion of hearing loss samples (with no restriction on types, severity, or the time of onset of hearing impairment); (6) articles with no restriction on cognitive status or other psychiatric disorder of subjects; and (7) those including hearing aids as an intervention in experimental groups. We excluded non-longitudinal studies and observational studies.

### Data extraction and analysis

Two independent authors (Yang Z and Teng Y) screened for the eligible studies and conducted data extraction. The corresponding author (Shi J) acted as an arbitrator for the final decision if a consensus could not be reached. Data from studies that investigated hearing loss in adults with different cognitive statuses or psychiatric disorders were grouped. Cognitive domains were subdivided as described in the study by Lezak et al. (2004): global cognition, episodic memory (delayed recall and immediate recall), executive function (attention, fluency, reasoning, and working memory), processing speed, semantic memory, and visuospatial ability. Language function was included later as a function of interest.

### Quality assessment

The Cochrane protocol for assessing the risk of bias (RoB2) was used for the quality assessment of RCTs. RoB2 consists of five domains: bias arising from the randomization process, bias due to deviations from intended interventions, bias due to missing outcome data, bias in the measurement of the outcome, and bias in the selection of the reported result (Sterne et al., 2019). The methodological index for non-randomized studies (MINORS) scale was used for the quality assessment of NRSIs, and the global ideal score was 16 for non-comparative studies and 24 for comparative studies (Slim et al., 2003; Zeng et al., 2015). The judgments were made by two independent authors (Yang Z and Ni J), and the corresponding author (Shi J) resolved the discrepancies, when needed.

### Sub-group analysis

Our study conducted a sub-group analysis according to subjects with different cognitive statuses and psychiatric disorders and different cognitive domains (where data were available).

### Statistical analysis

The meta-analysis was restricted to RCTs (Cuijpers et al., 2017). The standardized mean difference (SMD) with 95% confidence intervals (CIs) was chosen as the effect size to estimate the intervention effects of hearing aids on cognition in different domains, allowing for assessing the same outcome in various measurement ways. Our study obtained the standard deviation of the mean change score at the primary endpoint. If the required outcome metric was not reported in the primary study, values were calculated using available data. Before standardization, mean values were multiplied by −1 from some sets of studies to ensure that all scales pointed in the same direction, in which a positive SMD indicated a greater effect of hearing aid use on cognition (Andrade, 2020). Hedge's g values were used to assess clinical significance: 0.2 = small effect size, 0.5 = medium effect size, and 0.8 = large effect size. Hedge's g was calculated as follows: $g\;=\;\left({\bar{x}}_{1}-{\bar{x}}_{2}\right)/\sqrt{\left(\left(n_{1}-1\right)\;\times s1^{2}\;+\left(n_{2}-1\right)\times s2^{2}\right)/\left(n_{1}+n_{2}-2\right)}$, where ${\bar{x}}_{1}\;and\;{\bar{x}}_{2}$ were the sample means of each study, n1 and n2 were the sample sizes of each study, and s1^2^ and s2^2^ were the variances of each study.

Cochran's *Q* test and Higgins' *I*^2^ test were used to assess and quantify heterogeneity. *I*^2^ values of 25%, 50%, and 75% indicated low, moderate, and high degrees of heterogeneity, respectively (Sedgwick, 2015). The common-effect model was used when the *I*^2^ value was ≤ 50% based on the assumption that the same parameter underlying each study was reasonable. A random-effect model was used when the *I*^2^ value was > 50%. Publication bias was assessed by using Egger's test (Egger et al., 1997).

All statistical analyses were performed in RStudio, version 2022.02.2+485, and the meta-analysis was conducted using the meta package, version 5.2-0.

## Results

### Study identification and selection

**Figure 1:**
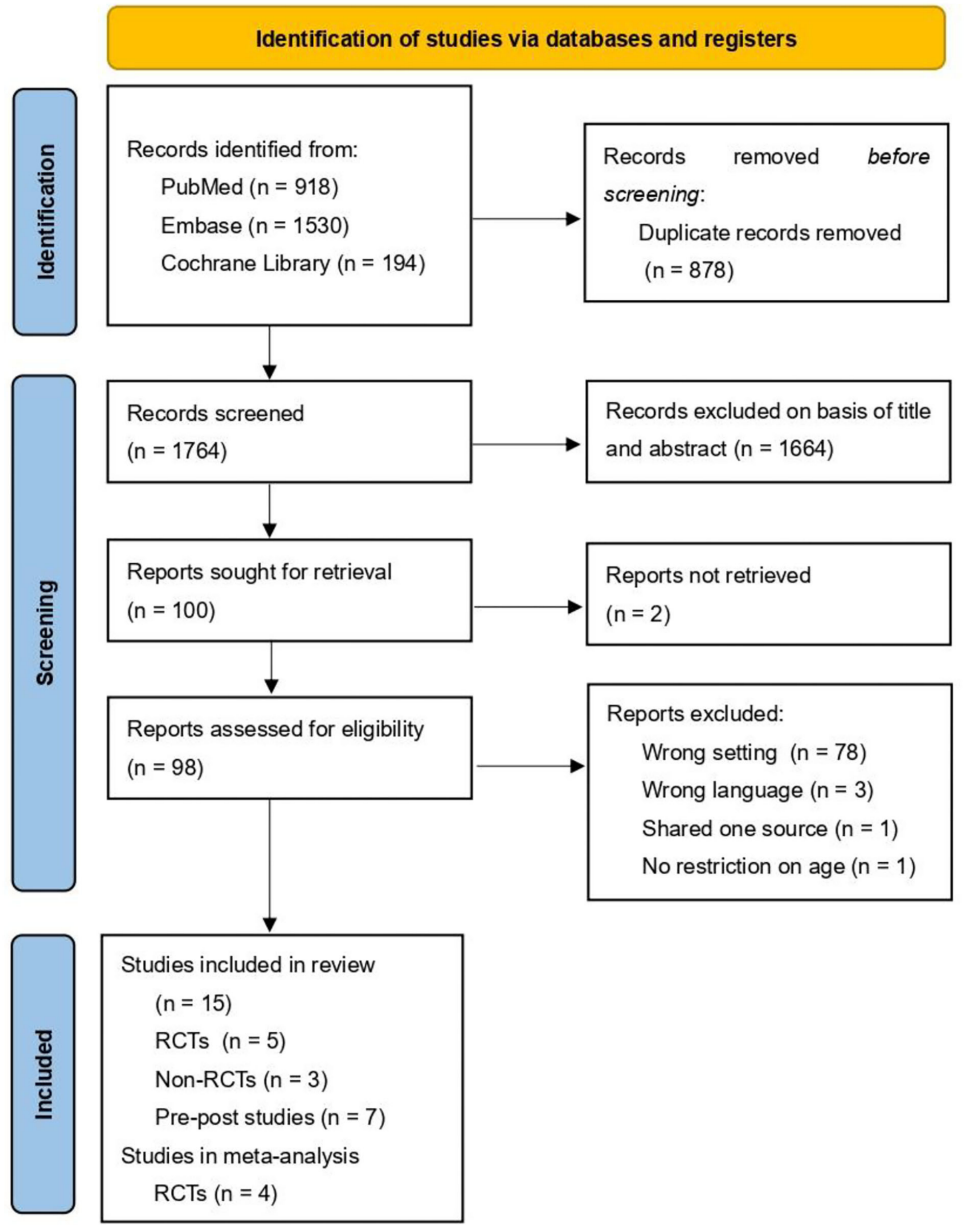
Preferred Reporting Items for Systematic Reviews and Meta-Analyses (PRISMA) flow diagram.

In the initial search, a total of 2,642 articles were reviewed from the three databases, of which 98 were retrieved for full-text screening. Eventually, 15 studies met the inclusion criteria, and four RCTs were included in a meta-analysis. Details of five RCTs and 10 NRSIs, including three non-RCTs and seven pretest–posttest studies, are demonstrated in Tables 1, 2 respectively. The search process is shown in Figure 1 according to the Preferred Reporting Items for Systematic Reviews and Meta-Analyses (PRISMA) statement (Page et al., 2021).

**Table 1:** Characteristics of RCTs.

**Ref**	**Country**	**Design**	**Population**	**Sample size^a^ and sex (n)**	**Mean age^b^**	**Baseline global cognition^b^**	**Baseline audiometric assessment^b^**	**Intervention and period**	**Cognitive test sessions**	**Outcome measures**	Changes from baseline*	
											**E**	**C**
Subjects without dementia												
(Mulrow et al., 1990)	USA	Parallel	Hearing impaired, > 64 years	E: 95, M/95 C: 99, M/99	E: 73 ± 7 C: 71 ± 5	SPMSQ ^d^: E: 0.47 ± 0.75 C: 0.18 ± 0.46	HFPTA: E: 53 ± 10 C: 51 ± 8	E: Hearing aids C: Blank 4 m	Baseline and 4 m	^#^ Global cognition: SPMSQ	−0.18 ± 0.71	0.1 ± 0.58
(Deal et al., 2017)	USA	Parallel	Bilateral hearing loss, 70–84 years	E: 20, M/5 C: 20, M/8	E: 77 ± 4.1 C: 78 ± 4.0	MMSE ≥ 23 for high school degree or less and ≥ 25 for college degree or higher	Three–frequency PTA (dB HL): E: 44 ± 6 C: 47 ± 10	E: Bilateral RIC hearing aids C: Successful aging intervention 6 m	Baseline and 6 m	Episodic memory: DWR	0.5 ± 1.2	1.1 ± 1.6
										Episodic memory: LMA	2.7 ± 2.9	0.7 ± 2.5
										Episodic memory: IL	0.8 ± 2.3	0.1 ± 1.9
										Language: WF	−0.1 ± 6.3	0.4 ± 5.1
										Language: BNT	0.5 ± 1.4	−0.1 ± 1.7
										Executive function: TMT–A	−2.8 ± 7.6	−1.3 ± 10.4
										Executive function: TMT–B	5.8 ± 31.1	−16.3 ± 28
										Processing speed: DSST	0.6 ± 5.6	1.2 ± 6.1
(Karawani et al., 2018)	USA	Parallel	Hearing impaired, 60–84 years	E: 18, M/8 C: 14, M/5	E: 75 ± 6.52 C: 74 ± 5.79	MoCA: E: 26.72 ± 1.77 C: 25.24 ± 2.45	PTA (0.5–4 kHz; dB HL): E: 42.58 ± 7.15 C: 40.12 ± 73.8 HF: E: 66.52 ± 11.88 C: 60.98 ± 13.78	E: RIC hearing aids C: Blank 6 m	Baseline and 6 m	^#^Working memory: LSWMT	8.2 ± 10.6	−2.3 ± 13.5
										Attention: FT	1.2 ± 12.2	6.5 ± 12.1
										Processing speed: PCPST	3.6 ± 17.8	2.3 ± 18.8
Subjects with Alzheimer's disease												
(Nguyen et al., 2017) ^f^	France	Double blind, Placebo, Crossover	Hearing loss Patients with AD, ≥ 65 years	E: 22, M/8 C: 26, M/11	E: 83 ± 6.2 C: 82.3 ± 7.2	ADAS–Cog: E: 18.1 ± 7.4 C: 19.0 ± 9.5	Hearing threshold (dB): E: 50.6 ± 11.4 C: 47.2 ± 9.6	E: Binaural hearing aids C: Placebo hearing aids 6 m	Baseline and 6 m	Global cognition: ADAS–Cog	1.8 ± 5.3	1.3 ± 5.3
										Global cognition: MMSE	−1.2 ± 2.9	−0.2 ± 4.1
										Anterograde memory: 16–iFCR	−1.88 ± 8.31	−1.21 ± 5.0
										Visual memory execution speed and attention: DST	0 ± 1.8	−0.4 ± 1.7
Subjects with depressive symptoms											Between group difference		
											MD	ES	t
(Brewster et al., 2022) ^g^	USA	Double blind, Parallel	Bilateral hearing loss, MDD or persistent depressive disorder, ≥ 60 years	E: 11, M/4 C: 14, M/4	E: 72.9 ± 9.2 C: 75 ± 6.5	MMSE: E: 29.5 ± 1 C: 28.4 ± 1.2	PTA: E: 38.8 ± 5 C: 44.3 ± 7.7	E: Active hearing aids C: Sham hearing aids 12 w	Baseline and 12 w	^#^ RBANS–H (immediate memory):	9.16	0.62	2.28
										RBANS–H (delayed memory)	−0.40	0.03	−0.12
										RBANS–H (attention)	3.53	0.23	0.60
										RBANS–H (language)	−4.36	0.25	−0.76
										RBANS–H (visuospatial/ constructional)	5.53	0.35	1.12
										Executive function: NIH Toolbox (DCCST)	6.88	0.36	1.16
										Executive function: NIH Toolbox (FT)	7.28	0.63	1.95

Ref, reference; a, intention–to–treat population (ITT population) except for research by Karewani, which conducted and matched the baseline data according to the subjects who completed the 6–month visits; b, Data are presented as mean ± standard deviation (SD). ^*^ Results were presented as changes from baseline, except for results of Brewster's study, which were between group mean differences and p–value. E, experimental group; C, control group; M, male subjects; d, the experimental group had a slightly worse Short Portable Mental Status Questionnaire (SPMSQ) score (p = 0.01), where a higher score indicated a worse impairment. ^#^, p–value < 0.05; HFPTA, high–frequency pure–tone average (average dB loss in the better ear at 1000, 2000, and 4000 Hz). m, month; PTA, pure–tone average hearing; RIC, receiver–in–canal; DWR, delayed word recall; LMA, logical memory A; IL, incidental learning; WF, word fluency (F, A, S); BNT, Boston Naming Test; TMT, Trail Making Test; DSST, Digit Symbol Substitution Test; MoCA, Montreal Cognitive Assessment; HF, high–frequency hearing (6–8 kHz; dB HL); LSWMT, NIH Toolbox List Sorting Working Memory Test; FT, NIH Toolbox Flanker Inhibitory Control and Attention Test (Flanker Test); PCPST, NIH Toolbox Pattern Comparison Processing Speed Test; f, data are collected at the 6–month visits only (before the crossover) as one of the primary endpoints relevant to both groups in this study was the change from the baseline of the Alzheimer's Disease Assessment Scale–Cognitive subscale (ADAS–Cog) after a 6–month period in both groups; AD, Alzheimer's disease; MMSE, Mini–Mental State Examination; 16–iFCR, 16–item Free and Cued Recall (sum of scores for the three cued recalls); DST, Digit Symbol Test; MDD, major depressive disorder; g, the study presented the data as between group difference; MD, mean difference; ES, effect size; w, week; RBANS–H, Repeatable Battery for the Assessment of Neuropsychological Status for Hearing Impaired Population, reported on cognitive domains assessed, rather than the name of measures in the original article; DCCST, NIH Toolbox Dimensional Change Card Sort Test; Shame hearing aids, devices programmed to a flat 30 dB HL.

**Table 2:** Characteristics of NRSIs.

**Ref**	**Country**	**Population**	**Sample size^a^ and sex (n)**	**Mean age^b^**	**Baseline global cognition^b^**	**Baseline audiometric assessment^b^**	**Intervention and Period**	**Cognitive test sessions**	**Outcome measures**	**Results** ^**b**^	
										**Pretest**	**Posttest**
**Characteristics of non–randomized controlled trials**											
(Tesch-Römer, 1997)^d^	Germany	Hearing impaired, 51–87 years	E: 70, M/30 C: 42, M/20	E: 71.8 ± 8.2 C: 71.5 ± 6.5	Mini–Mental State score: E: 1.1 ± 1.2 C: 0.7 ± 0.9	PTA (dB)^e^: E: 36.0 ± 9.0 C: 25.8 ± 8.7 PTA (dB)^f^: E: 47.3 ± 10.4 C: 37.8 ± 9.6	E: hearing aids C: blank 6 m	Baseline and 6 m	Speed: Digit symbol substitution	E: 40.1 ± 12.1 C: 43.1 ± 12.0	E: 41.0 ± 12.1 C: 43.6 ± 13.0
									Speed: digit letter	E: 106.5 ± 23.9 C: 109.3 ± 24.0	E: 109.3 ± 24.0 C: 113.7 ± 26.5
									Fluency: naming animals	E: 26.4 ± 7.6 C: 27.2 ± 7.3	E: 25.9 ± 7.6 C: 28.1 ± 8.7
									Fluency: letter “s”	E: 19.4 ± 7.1 C: 21.5 ± 7.8	E: 20.4 ± 8.3 C: 21.5 ± 7.1
									Vocabulary: spot–a–word	E: 19.5 ± 4.1 C: 20.4 ± 3.0	E: 19.8 ± 4.3 C: 21.2 ± 3.0
(van Hooren et al., 2005)^g^	the Netherlands	Hearing impaired, ≥ 60 years	E: 56, M/36 C: 46, M/29	E: 72.54 ± 7.30 C: 74.50 ± 6.77	MMSE: E: 27.91 ± 1.69 C: 27.96 ± 1.43	Hearing threshold (dB): E: 46.46 ± 7.30 C: 44.09 ± 7.69	E: hearing aids C: blank 1 y	Dual baseline (mean interval 13 d), and 1 y	Selective attention and speed of information processing: SCWT−12	E: 23.35 ± 6.45 C: 23.16 ± 5.01	E: 22.78 ± 2.10 C: 21.80 ± 2.10
									Selective attention and speed of information processing: SCWT–i	E: 31.67 ± 12.61 C: 37.23 ± 13.94	E: 37.22 ± 19.08 C: 35.75 ± 19.33
									Simple cognitive speed and cognitive flexibility: CST–ab	E: 30.50 ± 6.95 C: 31.39 ± 7.67	E: 29.08 ± 5.76 C: 29.43 ± 5.83
									Simple cognitive speed and cognitive flexibility: CST–i	E: 18.29 ± 13.81 C: 19.04 ± 15.57	E: 21.88 ± 15.49 C: 18.10 ± 15.67
									General information processing speed: LDST	E: 26.20 ± 6.41 C: 23.37 ± 6.78	E: 25.84 ± 3.29 C: 25.20 ± 3.39
									Intentional learning and verbal memory function: VVLT (immediate recall)	E: 20.95 ± 5.77 C: 20.61 ± 4.14	E: 25.61 ± 3.89 C: 25.30 ± 4.00
									Intentional learning and verbal memory function: VVLT (delayed recall)	E: 7.96 ± 3.04 C: 7.50 ± 2.90	E: 25.61 ± 3.89 C: 10.13 ± 2.24
									Semantic memory: VFT	E: 26.46 ± 6.16 C: 25.63 ± 7.39	E: 24.89 ± 5.24 C: 23.58 ± 5.36
(Doherty and Desjardins, 2015)^h^	USA	Hearing impaired, 50–74 years	E1: 11, M/NA C1: 8, M/NA	E1: 56.6 ± 3.4 C1: 55 ± 2.9	NA	NA	E1/2: hearing aids C1/2: blank 6 w	Baseline and 6 w	Working memory: Listening Span Test, *n*–back Test	Subjects in E1 and E2 both got significant improvement in auditory working memory, especially in noisy conditions, while subjects in control groups did not observe any changes in working memory tests.	
			E2: 13, M/NA C2: 8, M/NA	E2: 68.7 ± 4.1 C2: 67 ± 3.1						
Characteristics of pretest–posttest studies in within–subjects design											
Subjects with dementia											
(Allen et al., 2003)	UK	Hearing impaired, 67–96 years	31, M/6	84 ± 6.6	MMSE: 18.1 (mean)	Pure tone threshold of the better ear: 59.32 ± 9.66 dBHL	Hearing aids 24 w	Baseline, 4 w, 12 w, 24 w	Global cognition: MMSE	18.1	16.1
Subjects with mixed cognitive status											
(Acar et al., 2011)	Turkey	Hearing impaired, GDS: 6.8 ± 3.9 (mean ± SD) > 65 years	34, M/30	70.08 ± 4.8	MMSE: 20.38 ± 7.74 (mean ± SD)	Pure tone audiometric tests, mean hearing loss: right: 57.2, left: 56.3 dB	Hearing aids 3 m	Baseline and 3 m	^#^Global cognition: MMSE	20.38 ± 7.74	23.05 ± 7.59
(Magalhães and Iório, 2011)	Brazil	Hearing impaired, ≥ 60 years	50, M/27	NA	MSME: 21.6 ± 3.9 (mean ± SD)	Severe symmetrical bilateral hearing loss and an IPRF > 50%	Bilateral hearing aids 1 y	Baseline and 1 y	^#^Global cognition: MSME	21.6 ± 3.9	25.3 ± 3.3
Subjects with mixed cognitive status											
(Desjardins, 2016)^i^	USA	Hearing impaired, 54–64 years	6, M/NA	NA	NA	Air–conduction thresholds at octave frequencies: between 0.25 kHz and 8 kHz and at 6 kHz; bone–conduction thresholds at octave frequencies between 0.5 kHz and 2 kHz	Bilateral hearing aids 24 w	2 w, 4 w, 6 w, 12 w, 24w	Working memory: Listening Span Test	5/6 (83.3%)	
									Working memory: Reading Span Test	5/6 (83.3%)	
									Selective attention: the Coordinate Response Measure corpus	6/6 (100.0%)	
									Selective attention: the Stroop test	3/6 (50.0%)	
									Processing speed: Auditory Reaction Time Task	2/6 (33.3%)	
									Processing speed: the Digit Symbol substitution test	4/6 (66.7%)	
(Anzivino et al., 2019)^j^	Italy	Postlingually deafened, > 60 years	19, M/8	74.92 ± 5.4	MMSE ≥ 24/30	Left mean PTA: 48.21dB, Right mean PTA: 52.88 dB	Hearing aids 6 m	Baseline and 6 m	^#^Global cognition: MMSE	26.0 ± 1.03	27.66 ± 0.80
									^#^Episodic memory: RAVLT (immediate recall)	32.6 ± 3.80	36.6 ± 3.33
									^#^Episodic memory: RAVLT (delayed recall)	6.50 ± 1.14	8.16 ± 1.0
									Episodic memory: RAVLT (recognition correct)	12.16 ± 0.89	13.0 ± 1.19
									Episodic memory: RAVLT (recognition false)	1.6 ± 0.8	1.6 ± 0.4
									Working memory: Rey's figure recall	10.65 ± 2.74	6.66 ± 2.53
									Working memory: DSF	4.3 ± 0.4	4.5 ± 0.48
									Working memory: DSB	3.33 ± 0.51	2.8 ± 0.49
									Working memory: CSF	4.00 ± 0.3	4.33 ± 0.34
									Working memory: CSB	3.8 ± 0.38	3.83 ± 0.32
									Attention: MFTC (accuracy)	0.8 ± 0.03	0.91 ± 0.04
									Attention: MFTC (error)	1.00 ± 0.46	0.66 ± 0.34
									Attention: MFTC (time)	77.5 ± 14.9	76.16 ± 12.0
									Attention: TMT–A	92.07 ± 22.03	101.6 ± 27.97
									Attention: TMT–B	188.0 ± 35.67	202.8 ± 34.82
									Executive function: STI	25.08 ± 4.99	29.1 ± 6.03
									Executive function: STE	2.75 ± 2.64	4.33 ± 1.64
									Executive function: Rey's figure–copy	27.1 ± 1.71	24.0 ± 3.50
									Sematic memory: PVF	29.16 ± 4.45	27.6 ± 4.1
									Sematic memory: CSF	16.5 ± 2.1	17.6 ± 2.05
(Sarant et al., 2020)	Australia	Hearing loss, 60–84 years	98, M/45	72.5 ± 4.86	MMSE ≥ 24/30	Better ear PTA: 31.24 ± 7.9 dB (15–52.5 dB), Worse ear PTA: 38.44 ± 11.04 dB (17.5–97.5 dB)	Hearing aids 18 m	Baseline and 18 m	^#^Executive function: GML	58.81 ± 15.53	51 ± 15.35
									Psychomotor function: DET	2.58 ± 0.08	2.6 ± 0.08
									Working memory: ONB	2.96 ± 0.1	2.94 ± 0.08
									Attention: IDN	2.78 ± 0.06	2.78 ± 0.07
									Visual learning: OCL	0.94 ± 0.14	0.96 ± 0.11
Subjects with depressive symptoms											
(Boi et al., 2012)	Italy	Hearing impaired, depressive symptoms, ≥ 70 years	15, M/10	78.00 ± 4.40	MMSE ≥ 24/30	14 patients: 56–70 dB 1 patient: 71–90 dB	Binaural hearing aids 6 m	Baseline, 1m, 3 m, 6 m	^#^Global cognition: MMSE	26.93 ± 3.10	28.17 ± 2.17
									^#^Executive and visuospatial function: CDT	1.93 ± 1.08	1.93 ± 0.93

Ref, reference. a, number of subjects at the baseline; b, Data are presented as mean ± SD; E, experimental group; C, control group; d, we only presented and analyzed one of the controlled groups that included hearing impaired subjects; M, male subjects; PTA, pure–tone average hearing; e, PTA (0.5, 1, 2kHz); f, PTA (1, 2, 4 kHz); m, month; g, the results in this study are presented as mean ± SE, and we got the SDs in Excel by the equation, SD = SE ^*^ sqrt (N) according to Cochrane Handbook for Systematic Reviews of Interventions; MMSE, Mini–Mental State Examination; y, year; SCWT, Stroop Color–Word Test; SCWT−12, last part time subtracted from the mean score for the first and second parts; SCWT–i, interference score; CST, Concept Shifting Task; CST–ab, the mean score of the first and second parts; CST–i, the additional time needed to shift between both sets of stimuli; LDST, Letter–Digit Substitution Test; VVLT, Visual Verbal Learning Test; VFT, Verbal Fluency Test; h, in this study, authors did not mention the baseline cognitive or psychiatric status of subjects; baseline audiometric status and outcomes were presented as bar charts, thus, the outcomes in our table were described qualitatively, rather than quantitatively; E1/C1, middle–aged subjects; E2/C2, young to older subjects; NA, not applicable; w, week; GDS, GDS–short form with a cutoff score of 7; Scores of > 7 indicate the presence of depression; IPRF, Speech Recognition Percent Index; #, p–values < 0.05; MSME, Mental State Mini Exam; i, in this study, all subjects passed the Short Portable Mental Health Status Questionnaire, but the exact scores were not presented; the effects were measured in each single subject without synthesis; j, in this study, subjects were divided into cochlear implantation group and hearing aids group, and we only collected the data at 6–month visit in hearing aids group; RAVLT, Rey Auditory Verbal Learning Task; Rey's figure, Rey–Osterrieth Complex Figure Test; DSF, Digit Span Forward; DSB, Digit Span Backward; CSF, Corsi Span Forward; CSB, Corsi Span Backward; MFTC, Multiple Features Target Cancellation; TMT, Trail Making Test; STI, Stroop Test Interference; STE, Stroop Test Errors; CSF, Categorical Semantic Fluency; PVF, Phonological Verbal Fluency Task; DET, detection test; IDN, identification test; ONB, One Back Test; OCL, One Card Learning test; GML, Groton Maze Learning test; CDT, Clock Drawing Test.

### Randomized controlled trials

#### Characteristics of RCTs

A total of five RCTs were included, of which four were conducted in the United States (Mulrow et al., 1990; Deal et al., 2017; Karawani et al., 2018; Brewster et al., 2022) and one was in France (Nguyen et al., 2017). Aside from the research by Mulrow et al. (1990) published in 1990, the rest of them were published between 2017 and 2022.

Of all evaluated RCTs, two were experimental, placebo-controlled, double-blind studies (Nguyen et al., 2017; Brewster et al., 2022) and three were experimental, controlled, parallel, randomized studies (Mulrow et al., 1990; Deal et al., 2017; Karawani et al., 2018). A total of 339 subjects were included in the systematic review who were aged ≥ 60 years. In total, three studies recruited 266 subjects without a confirmed diagnosis of dementia (Mulrow et al., 1990; Deal et al., 2017; Karawani et al., 2018), measured by the Mini-Mental State Examination (MMSE) (Deal et al., 2017), Montreal Cognitive Assessment (MoCA) (Karawani et al., 2018), and Short Portable Mental Status Questionnaire (SPMSQ) (Mulrow et al., 1990), respectively. In the research by Mulrow et al. (1990), 23% of the subjects were identified with depression (Geriatric Depression Scale > 5) and 1% was significantly cognitively impaired (SPMSQ > 2). A previous study investigated 48 patients with Alzheimer's disease (AD) in accordance with the Diagnostic and Statistical Manual of Mental Disorders (DSM IV) and the National Institute of Neurological and Communicative Disorders and Stroke–Alzheimer's Disease and Related Disorders Association (NINCDS-ADRDA) criteria, with MMSE scores between 10 and 28 (Nguyen et al., 2017). Another study recruited 25 patients with major depressive disorder or persistent depressive disorder (Brewster et al., 2022), while all included patients had normal global cognition measured by the MMSE. All RCTs used hearing aids as the intervention with a duration ranging from 12 weeks to 6 months. The research by Mulrow et al. (1990) recruited only male subjects, while the other four studies recruited both male and female subjects. The exact types of hearing aids were not presented in our study.

For subjects without dementia, the use of hearing aids resulted in significant improvements in global cognition (measured by SPMSQ) (Mulrow et al., 1990) and working memory (measured by the NIH Toolbox List Sorting Working Memory Test) (Karawani et al., 2018). By contrast, in the research by Nguyen et al. (2017), in patients with AD, no significant improvement was found in global cognition (measured by the ADAS-Cog and MMSE), anterograde memory (measured by the 16-item Free and Cued Recall), and visual memory execution speed and attention (measured by the Digit Symbol Test). For patients with depressive disorder, significant improvement was noted in immediate memory (measured by the RBANS) (Brewster et al., 2022). In the research by Deal et al. (2017), the authors did not make a comparison between the experimental and control groups.

#### Meta-analysis of RCTs

**Figure 2:**
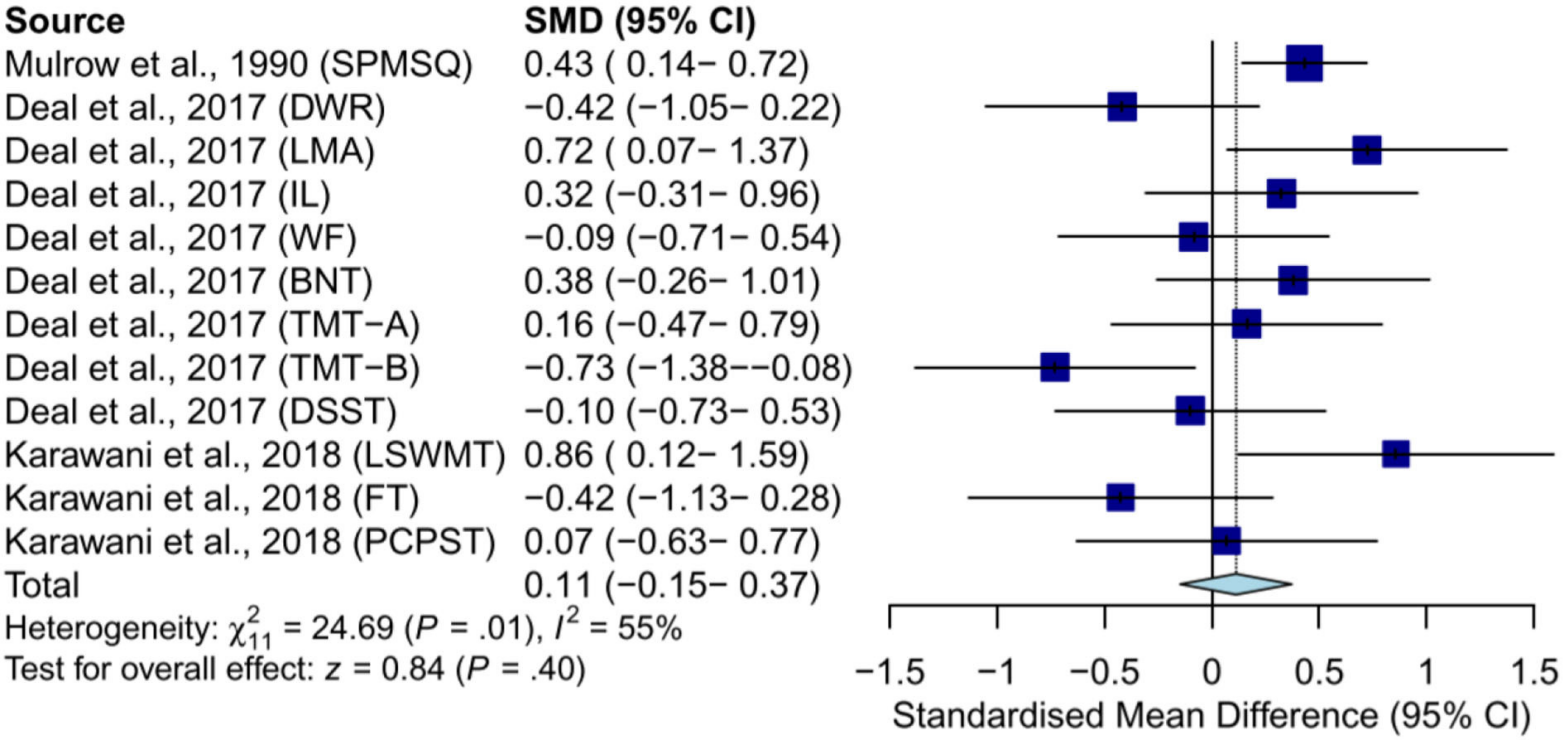
Meta-analysis of SMD (random-effect model) in effects of hearing aids on cognition in subjects without dementia. SPMSQ, Short Portable Mental Status Questionnaire; DWR, delayed word recall; LMA, logical memory A; IL, incidental learning; WF, word fluency (F, A, S); BNT, Boston Naming Test; DSST, Digit Symbol Substitution Test; LSWMT, List Sorting Working Memory Test; FT, Flanker Test; PCPST, Pattern Comparison Processing Speed Test.

As for meta-analysis, our study found no statistically significant effect of hearing aid use on cognitive function in subjects without dementia [random-effect model: SMD = 0.11, 95% CI: −0.15–0.37, *p* = 0.40; (heterogeneity: χ^2^ = 24.69, df = 11, *p* = 0.01, *I*^2^ = 55%)] (Figure 2). Studies in patients with major depressive disorder were not included in the meta-analysis because they did not report usable data.

#### Subjects without dementia

A previous study examined the effects of hearing aid use on episodic memory (measured by delayed word recall, logical memory A, and incidental learning, respectively) and found that hearing aids were not significantly more effective than the control condition where subjects received successful aging intervention at a 6-month follow-up (random-effect model: SMD = 0.21, 95% CI: −0.45–0.86, *p* = 0.53; [heterogeneity: χ^2^ = 6.25, df = 2, *p* = 0.04, *I*^2^ = 68%]) (Supplementary Figure S1) (Deal et al., 2017).

A study examined the effects of hearing aid use on language (measured by word fluency (F, A, S) and Boston Naming Test) and found that hearing aids were not significantly more effective than the control condition where subjects received successful aging intervention at a 6-month follow-up [common-effect model: SMD = 0.14, 95% CI: −0.30–0.59, *p* = 0.53; (heterogeneity: χ^2^ = 1.04, df = 1, *p* = 0.31, *I*^2^ = 4%)] (Supplementary Figure S2) (Deal et al., 2017).

Furthermore, two studies examined the effects of hearing aid use on general executive function, including executive function (measured by the Trail Making Test (TMT) part A and B), processing speed (measured by the Digit Symbol Substitution Test and Pattern Comparison Processing Speed Test), attention (measured by the Flanker Test), and working memory (measured by the List Sorting Working Memory Test) and found that hearing aids were not significantly more effective than the control condition where subjects received the successful aging intervention or no treatment at 6-month follow-up (random-effect model: SMD = −0.05, 95% CI: −0.33–0.22, *p* = 0.71; [heterogeneity: χ^2^ = 11.73, df = 5, *p* = 0.04, *I*^2^ = 57%]) (Supplementary Figure S3) (Deal et al., 2017; Karawani et al., 2018).

For further research, the sub-group analysis of single executive function was conducted; the results showed that hearing aids were not significantly more effective on executive function (SMD = −0.27, 95% CI: −0.72–0.18) and processing speed (SMD = −0.02, 95%CI: −0.49–0.44) (Supplementary Figure S3). The results of working memory and attention were not generated because of the limited number of studies.

#### Subjects with Alzheimer's disease

**Figure 3:**
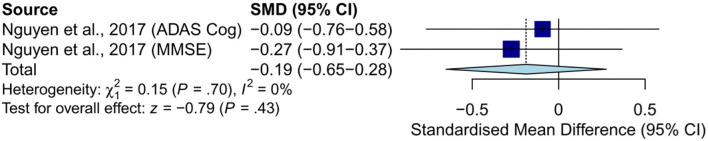
Meta-analysis of SMD (common-effect model) in the effects of hearing aids on global cognition in subjects with AD. ADAS-Cog, Alzheimer's Disease Assessment Scale-Cognitive subscale; MMSE, Mini-Mental State Examination.

Only one study examined the effects of hearing aids on global cognition in subjects with AD and found that hearing aids were not significantly more effective than the placebo control condition at a 6-month follow-up (common-effect model: SMD = −0.19, 95% CI: −0.65–0.28, *p* = 0.43; [heterogeneity: χ^2^ = 0.15, df = 1, *p* = 0.70, *I*^2^ = 0%]) (Figure 3) (Nguyen et al., 2017). The results of anterograde memory and visual memory execution speed and attention were not generated because of the limited number of studies.

#### Risk of bias

**Figure 4:**
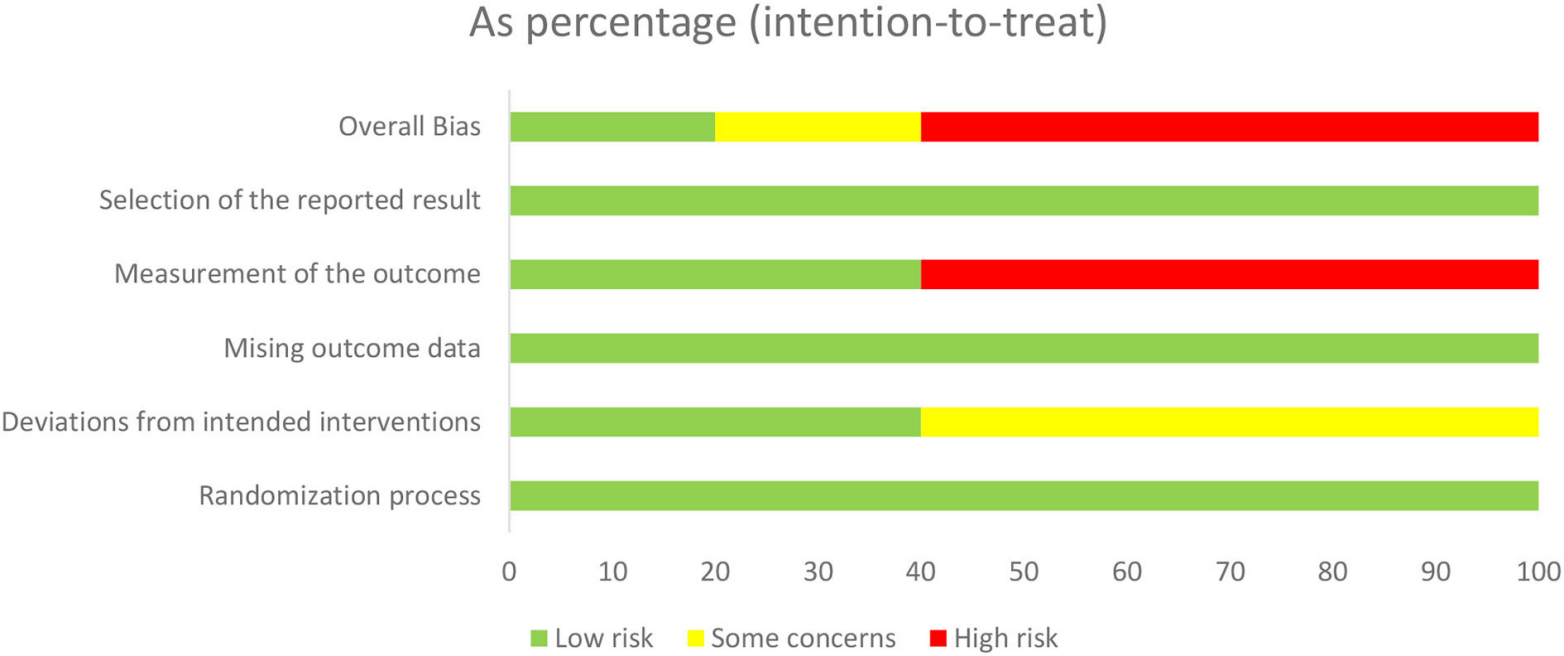
Risk of bias of the included RCTs.

**Figure 5:**
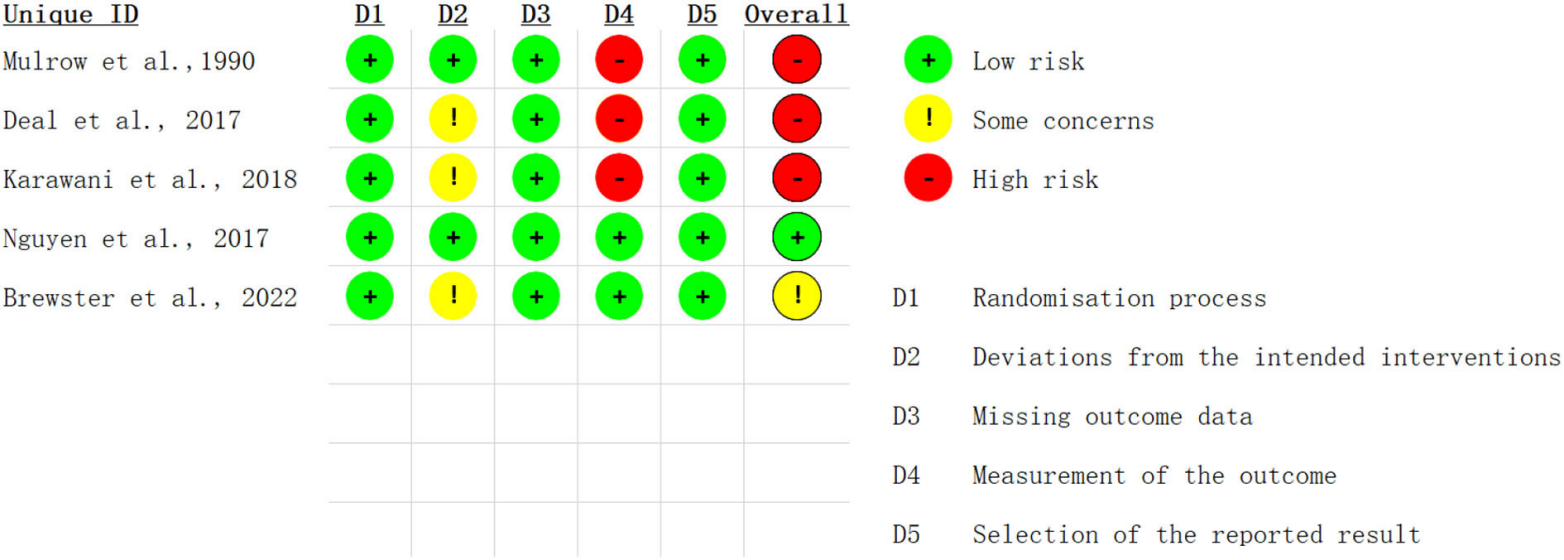
Risk of bias assessment summary.

The risk of bias of the included RCTs is shown in Figures 4, 5. All the included RCTs showed low risk bias due to the randomization process, missing outcome data, and selection of the reported result. As for bias from intended interventions, 60% showed some concerns and 40% showed low risk. Regarding bias in the measurement of the outcome, 60% showed high risk and 40% showed low risk. As for overall bias, 60% showed high risk, 20% showed some concerns, and the rest showed low risk.

### Characteristics of NRSIs

#### Characteristics of non-randomized controlled trials

Of three non-RCTs that were included, one was conducted in Germany (Tesch-Römer, 1997), one was conducted in the Netherlands (van Hooren et al., 2005), and the other was conducted in the United States (Doherty and Desjardins, 2015), published in 1997, 2005, and 2015, respectively.

From these three non-RCTs, our study included 254 subjects aged > 50 years. All studies reported matched demographic data between the intervention groups and the control groups at baseline, except for the research by Tesch-Römer (1997), where the intervention group subjects showed worse hearing impairment. Subjects in the studies by Tesch-Römer (1997) and van Hooren et al. (2005) had normal cognition measured by Mini-Mental State score or MMSE, and the intervention groups received hearing aids, while the control groups with hearing loss received no aural rehabilitation, with a period of 6 months and 1 year, respectively. The research by Doherty and Desjardins (2015) did not mention the baseline cognitive or psychiatric status of subjects, and the intervention groups received hearing aids for 6 weeks, while the control groups received no aural rehabilitation.

No statistically significant improvement in cognitive function was found in the studies by Tesch-Römer (1997) and van Hooren et al. (2005), including attention, processing speed, semantic memory, and the rest of the other domains. In addition, the research by van Hooren et al. (2005) reported a poorer performance on the Stroop Color–Word Test-12 in the intervention group after 1 year of hearing aid use (*p* = 0.02). However, in the research by Doherty and Desjardins (2015), subjects with hearing aids showed significant improvement in auditory working memory, especially in noisy conditions, while the control groups did not demonstrate any changes in working memory performance.

#### Characteristics of pretest–posttest studies

The selected pre- and post-intervention studies were conducted in six different countries: Italy (*n* = 2) (Boi et al., 2012; Anzivino et al., 2019), Turkey (*n* = 1) (Acar et al., 2011), Brazil (*n* = 1) (Magalhães and Iório, 2011), the United States (*n* = 1) (Desjardins, 2016), the United Kingdom (*n* = 1) (Allen et al., 2003), and Australia (*n* = 1) (Sarant et al., 2020), and they were published between 2003 and 2020.

From these seven pre- and post-intervention studies, 253 subjects aged ≥ 54 years were included, and all studies made a pre- and post-intervention comparison using a within-subject design. Regarding pre-intervention global cognition, there were three studies including 123 subjects with normal global cognition according to the MMSE or the Short Portable Mental Health Status Questionnaire (Desjardins, 2016; Anzivino et al., 2019; Sarant et al., 2020), two studies including 84 subjects with mixed cognitive status measured by the MMSE or Mental State Mini Exam (MSME) (Acar et al., 2011; Magalhães and Iório, 2011), and one study including 31 subjects with dementia (Allen et al., 2003). Regarding mental status, there was one study including 15 subjects with depressive symptoms (Boi et al., 2012) and one study including subjects with depression (Acar et al., 2011). The subjects received hearing aids for 3–18 months. Compared with RCTs and non-RCTs, some included pretest–posttest studies had multiple cognitive test sessions (Table 2) (Allen et al., 2003; Boi et al., 2012; Desjardins, 2016). The cognition domains mentioned included global cognition, visuospatial ability, episodic memory (delayed recall and immediate recall), attention, executive functions, processing speed, semantic memory, language, working memory, and visual learning (Table 2), and there were a total of 30 screening tests conducted in all groups of the included population, and eight screening tests showed significant improvements in cognitive functions after the use of hearing aids, excluding the research by Desjardins (2016), where the author did not generate the results of each subject. Subjects with mixed cognitive statuses achieved performance improvement in the global cognition test (measured by the MMSE or MSME). Subjects with normal global cognition achieved performance improvement in the global cognition test (measured by the MMSE), episodic memory (measured by the Rey Auditory Verbal Learning Task), and executive function (measured by the Groton Maze Learning Test). Subjects with depressive symptoms achieved performance improvement in the global cognition test (measured by the MMSE) and executive and visuospatial function (measured by the Clock Drawing Test).

#### Quality assessment of NRSIs

The MINORS index was applied to three non-RCTs with 19 scores, 19 scores, and 15 scores, respectively. The MINORS index was applied to 7 pretest–posttest studies using a within-subject design, with scores ranging from 9 to 12 (Supplementary Table S2).

## Discussion

To the best of our knowledge, this study is the first systematic review and meta-analysis that sheds light on hearing aids and cognitive functions in middle-aged and older subjects with different cognitive statuses and psychiatric disorders, and the first meta-analysis trying to make a data synthesis of different cognitive functions. Compared with previous systematic reviews of Sanders et al. (2021) or Mamo et al. (2018) that paid attention to one certain type of subject or of Utoomprurkporn et al. (2020) that only analyzed global cognitive function, our review paid attention to three specific groups of subjects and varieties of cognitive functions. In the systematic review, results were mixed. In subjects without dementia or with normal global cognition, the use of hearing aids improved cognitive performance in some tests of global function, working memory, and executive function. In subjects with mixed cognitive statuses, the use of hearing aids improved their cognitive performance in tests of global function. In subjects with AD or dementia, no improvement was found in all cognitive tests. Our study also found that in subjects with depressive symptoms, the use of hearing aids improved cognitive performance in some tests of immediate memory, global cognition, and visuospatial function. However, compared with these significant improvements, there were more tests that were not improved at all in all kinds of subjects with different cognitive statuses or psychiatric disorders, including language, attention, and processing speed. When restricted to RCTs, the meta-analysis indicated that the use of hearing aids had no significant effects on cognitive function, including episodic memory, language, and executive function, in subjects without dementia, and that the use of hearing aids had no significant effects on global cognition in subjects with AD.

### Hearing aids in subjects with AD or dementia

The negative effects found in subjects with AD or dementia were similar to the results of Bucholc et al. (2022), where the deterioration of cognition in patients with AD was only delayed, rather than impeded, despite the use of hearing aids. As AD is the most common type of dementia (Jia et al., 2020), we speculated that the limited effectiveness of hearing aids in patients with AD or dementia is attributed to factors including the specific mechanism underlying AD and hearing loss and the choice of an endpoint. First of all, most patients with AD underwent degeneration and atrophy of neurons and a series of pathological progression at the early stages, predating cognitive decline for many years. In addition, hearing loss and AD shared common causes. Mitchell et al. (2020) have found a significant genetic overlap but not a causal relationship between hearing loss and AD. Age-related vascular pathology or cerebrovascular disease is detrimental to both sensory input and cognition (Eckert et al., 2013; Livingston et al., 2020). In addition, Golub et al. (2021) found that hearing loss was associated with β-amyloid burden by positron emission tomography (PET) scans. Irace et al. (2022) noted the relevance between hearing loss in the left ear and β-amyloid burden. It is hard to determine which of those two impairments, hearing loss or AD, comes first; thus, a hearing aid might not be an appropriate therapeutic method because the development trajectory or causal relationship between hearing loss and AD is unclear. Furthermore, we speculated that, in AD or dementia patients with hearing loss, cognitive decline is attributed not only to decreased sensory input but also to the deteriorated ability of information processing and output, which cannot be modified by hearing aids as they amplify sound directly and work on peripheral hearing loss, instead of impaired speech perception (Gates et al., 2002). In addition, just like the research by Neff et al. (2019), no evidence supported that hearing aids influenced dementia neuropathology. Regarding the endpoints, both the included studies chose the MMSE or ADAS-Cog as the assessments. Although the MMSE and ADAS-Cog were relatively reliable and the most common available measures in the ADNI study, neither of them were sensitive when calculating the minimum detectable effect size in change from baseline. Functional scores or composite assessments should be put into use, and the trial design should involve a longer period (Huang et al., 2015; Evans et al., 2018).

### Hearing aids in subjects without dementia or with depressive symptoms

Unfortunately, similar to the results of Sanders et al. (2021), our study cannot draw a definite conclusion about whether using hearing aids could improve cognitive functions in subjects without dementia due to conflicting results. There are multiple reasons underlying the conflicting results.

A probable significant contributor to the conflicts is a random error mostly due to the small sample size as the included studies hardly had a sample size of > 100, which might have led to high false-positive rates. This is an inevitable problem and also hard to manage because it is currently difficult to estimate the minimum sample size in these kinds of studies as the exact effects of hearing aids are unclear. The study design, recruitment of the study population, and cognitive screening process might be other causes for the conflicting results.

The high risk of bias of RCTs in the measurement of outcome was owing to a lack of blind design as cognitive assessments would be influenced if outcome assessors were aware of the intervention received by study participants. As a result, despite the difficulty in placebo control settings of hearing aids, as sham hearing aids were hard to conceal, it is important to find a better way of placebo and blind settings in the future. It also should be mentioned that the study by Deal et al. (2017) was a feasibility pilot trial setting stages for the National Institute on Aging-funded ACHIEVE trial, where only within-subjects pre-/post-comparisons were made. Our study regarded it as an RCT; thus, the results in the meta-analysis might be influenced. Aside from RCTs, almost half of the included studies were pretest–posttest studies with within-subject designs, thus making it difficult to draw a reliable conclusion about whether there was an actual benefit of hearing aids on cognition without a control group, owing to high heterogeneity this study type might involve (Coalition for Evidence-Based Policy, 2007). In addition, the intervention time of the included studies ranged from 3 months to 18 months, which possibly resulted in negative therapeutic results because of early discontinuation to obtain benefits (Guyatt et al., 2011).

The different inclusion criteria in the included studies might also have led to diverse results. Some studies included subjects with mixed cognitive statuses; thus, the inconsistency of cognitive abilities of the study population might have led to selection bias or misclassification. In addition, the damage on each cognitive domain remained unadjusted at baseline in each study because it was difficult to set the inclusion criteria based on cognitive impairments in specific domains and degree of damage. If the subjects did not have substantial cognitive dysfunction, the results would have reduced sensitivity to improvement. In addition, the diverse definitions of hearing loss also made it challenging to interpret the results since different categorizations and degrees of hearing loss might represent different levels of impairment and ability decline (Powell et al., 2021b).

The cognitive screening process might matter as well. People with hearing loss tend to obtain a lower score in standard orally administered cognitive tests like MoCA (Utoomprurkporn et al., 2020). The significant effects of hearing aids on global cognition achieved in pretest–posttest studies are likely due to the improved scores in certain hearing-dependent subtests of the screening tools (Vasil et al., 2021), as a result of the instant improvement in hearing ability. This result is in line with the results of MacDonald et al. (2012) study, which noted significant improvements in the MMSE after hearing augmentation in the elderly. Hence, a modified visually cognitive assessment and its cutoff point should be validated for cognitive tests in the population with hearing loss (Utoomprurkporn et al., 2021). As for the disagreements among the sub-domains of cognition, the characteristics of each tool and different aspects of cognitive function might be responsible. For example, delayed recall is more sensitive than immediate recall in distinguishing between subjects with mild cognitive decline and normal cognition (Tian et al., 2003; Takayama, 2010), meaning that in the population without dementia, the subjects are more likely to get a lower score in delayed recall, thus leading to a larger effect estimate and a positive result. Except for sensitivity, the performance of the TMT is associated with the education level and age (Wei et al., 2018), while that of the 60-item Boston Naming Test is not (Serrano et al., 2001). In addition, the SPMSQ was not originally developed to detect changes in function (Pfeiffer, 1975). These differences among each screening tool should not be ignored in further research. In addition, some tools, such as RBANS-H, are specifically established for patients with hearing loss; thus, the results will not be influenced by auditory skills (Claes et al., 2016). Comparatively, other auditory-dependent screening tools might lead to different performances due to sensory loss (Füllgrabe, 2020; Nichols et al., 2022). Another factor is the learning effects of neuropsychological tests. Since some pretest-posttest studies conducted multiple cognitive tests in the process, there was a reasonable doubt that it was the learning effects leading to significant improvements in cognitive performance (Hijman et al., 1992). Except for cognitive impairment, hearing loss might promote progress in certain cognitive function such as visuospatial abilities. Utoomprurkporn et al. (Utoomprurkporn et al., 2022) found that patients with mild cognitive impairment and hearing loss performed as well as cognitively healthy subjects without hearing loss in tests of visuospatial abilities, thus reminding us to pay more attention to different aspects of cognitive functions instead of one certain domains when testing patients with cognitive impairment and hearing loss.

Apart from the issues listed before, another factor that contributed to mixed results is that the link between hearing loss and cognitive decline cannot be determined in each subject as no single hypothesis can explain it. Multiple reasons might underlie the association between hearing loss and cognitive impairment, such as the sensory deprivation hypothesis and information degradation hypothesis (Powell et al., 2021a). The sensory deprivation hypothesis suggests that prolonged hearing loss would cause a detrimental effect to the brain structure, including reduced cortical brain volume (Eckert et al., 2019), temporal lobe volume (Armstrong et al., 2019), frontal cortex, and hippocampus (Uchida et al., 2018; Rudner et al., 2019), as well as gray matter density and white matter integrity (Lin et al., 2014; Croll et al., 2020); thus, the impaired brain structures cause a cognitive decline, such as semantic memory, speech understanding, and processing speed. Comparatively, the information degradation hypothesis assumes that with the degradation of hearing input, increased cognitive processing is needed to compensate; thus, higher cognitive demands required reduce cognitive reserve available for other tasks, such as working memory (Tun et al., 2009; Peelle, 2018). It suggests a temporary cognitive impairment cause, which is likely to be restored by the amelioration of sensory input; this could be a possible explanation for the improvements in executive functions according to our review. Hence, as the mechanism between hearing loss and cognitive decline remains unclear, the subjects recruited in each trial might undergo different types and onset of cognitive decline associated with hearing loss.

In addition, our study researched the effects of hearing aids on subjects with depressive symptoms separately as depression has been recognized as a contributor to hearing loss and dementia and is likely to be influenced by the use of hearing aids itself (Livingston et al., 2017). The interaction between hearing aids and depression, as well as the association between depression and cognitive decline, might contribute to the mixed results; thus, further research on the effects of hearing aids on depression is needed.

Despite the limited effects of hearing aids on cognitive function, the Lancet Commission encouraged the use of hearing aids for hearing loss (Livingston et al., 2020). It should be pointed out that hearing aids do help with depressive symptoms, loneliness symptoms, and mental health quality of life in older adults with hearing loss, regardless of its effects on cognitive function (Choi et al., 2016; Contrera et al., 2016, 2017).

## Limitations

Our study had several limitations. First, on account of the limited number of RCTs, our study included both RCTs and NRSIs; thus, considerable heterogeneity caused by different types of study design remained across most outcomes. The number of studies included in our meta-analysis was limited, which made it inappropriate to draw a definite conclusion and left the problem still unsolved. Furthermore, our study did not conduct tests of publication bias since funnel plots or Egger's tests are only inspected when at least 10 trials are included. Hence, our study could not evaluate the publication bias. In addition, although our study focused on the middle-aged and older population with hearing loss, no restriction was set on the types and time of onset of hearing loss for the inclusion criteria. Since the research by Alattar et al. (2020) revealed that the severity of hearing loss was associated with the worse performance of the MMSE and TMT-B, the confounding factors might make it hard to discover the reason behind it.

## Conclusion

For subjects without dementia, hearing aids might improve cognitive test performance in specific cognitive domains, such as executive function. The exact effect of hearing aids on cognitive function in subjects with depressive symptoms remains unclear. No significant improvement of hearing aids on cognitive function was found in middle-aged and older hearing loss adults with AD or dementia. Long-term, well-designed RCTs and well-matched comparison-group studies with large sample sizes and specific target populations are necessary for validation.

## Data availability statement

The original contributions presented in the study are included in the article/Supplementary material, further inquiries can be directed to the corresponding authors.

## Author contributions

ZY, JN, JS, and JT contributed to the study design. ZY, JN, YT, MS, and JS contributed to statistical analysis and interpretation of data. ZY, YT, and MS contributed to the drafting of the manuscript. JN, MW, TLi, DF, TLu, HX, WZ, JS, and JT contributed to the critical revision of the manuscript for important intellectual content. All authors contributed to the article and approved the submitted version.

## Funding

This study was supported by the fundamental research funds for the central universities (2019-JYB-TD-007), Qihuang Scholar Foundation (China), and the collaborative research project of Beijing University of Chinese Medicine (BZY-JMZY-2022-002).

## Conflict of interest

The authors declare that the research was conducted in the absence of any commercial or financial relationships that could be construed as a potential conflict of interest.

## Publisher's note

All claims expressed in this article are solely those of the authors and do not necessarily represent those of their affiliated organizations, or those of the publisher, the editors and the reviewers. Any product that may be evaluated in this article, or claim that may be made by its manufacturer, is not guaranteed or endorsed by the publisher.
