# Supplementary material for: Effect of hearing aids on cognitive functions in middle-aged and older adults with hearing loss: A systematic review and meta-analysis
Source: Front Aging Neurosci. 2022 Nov 14;14:1017882. doi: 10.3389/fnagi.2022.1017882 (PMC9704725; doi:10.3389/fnagi.2022.1017882)
Supplement: Supplementary file 1 [file Data_Sheet_1.docx]

Supplementary Material

# Supplementary Figures

**Supplementary Figure S1. Meta-analysis (random-effect model) of effects of hearing aids use on episodic memory in subjects without dementia.**


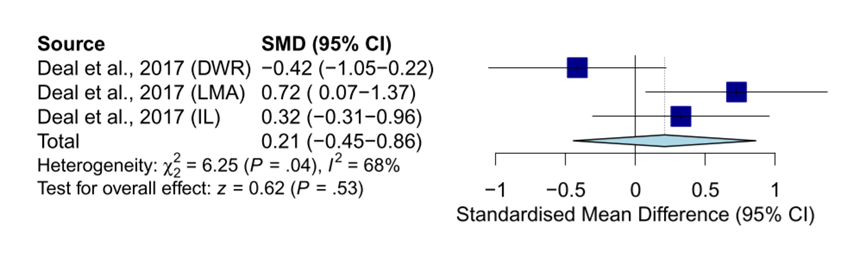


Abbreviation: DWR: delayed word recall. LMA: logical memory A. IL: incidental learning.

**Supplementary Figure S2. Meta-analysis (common-effect model) of effects of hearing aids use on language in subjects without dementia.**


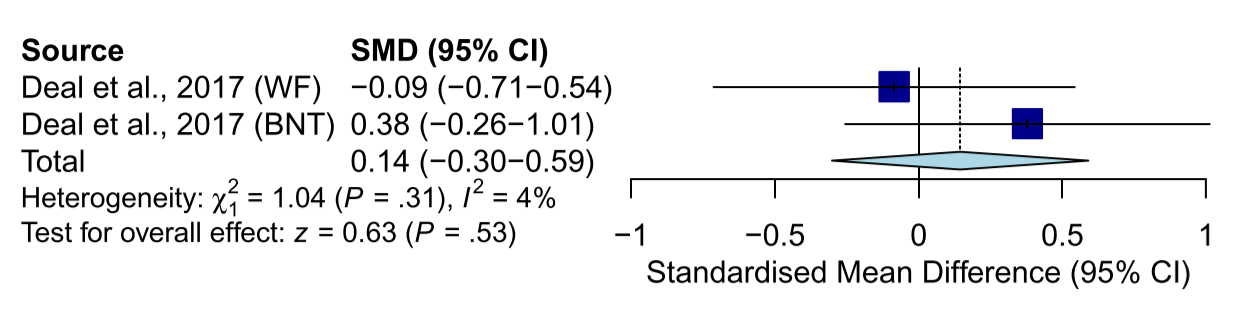


Abbreviation: WF: word fluency (F, A, S). BNT: Boston Naming Test.

**Supplementary Figure S3. Meta-analysis (random-effect model) of effects of hearing aids use on single executive function in subjects without dementia.**
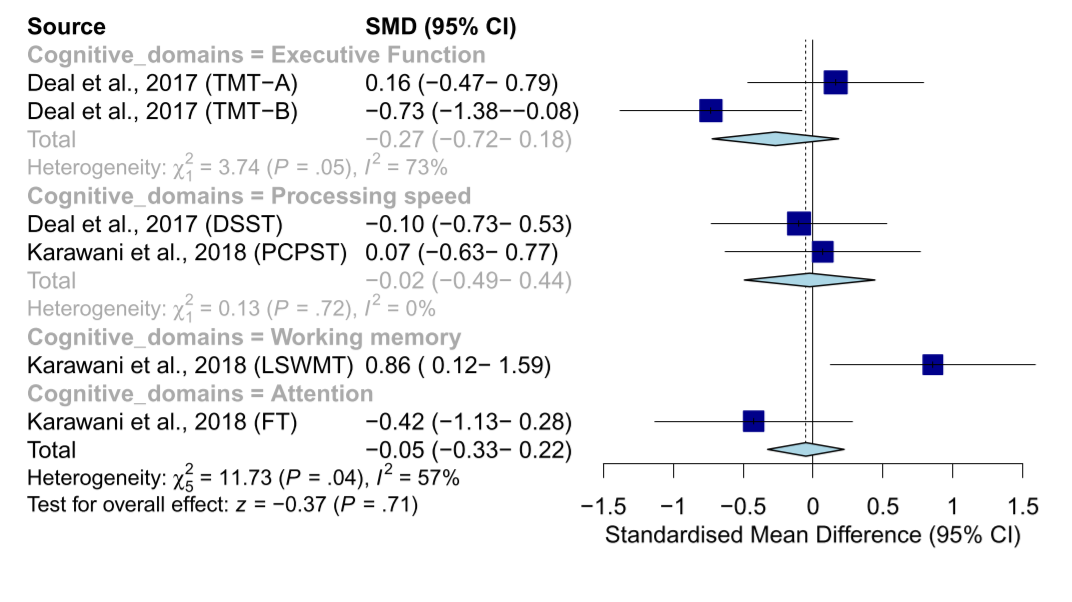


Abbreviation: TMT-A/B: Trail Making Test A/B. DSST: Digit Symbol Substitution Test. PCPST: Pattern Comparison Processing Speed Test. LSWMT: List Sorting Working Memory Test. FT: Flanker Test.

# Supplementary Tables

**Supplementary Table S1. Search terms and results.**

| **PubMed** | |
| --- | --- |
| **Terms** | **Total** |
| ((hearing aids[MeSH Terms]) OR (hearing aids[Title/Abstract])) AND (((Alzheimer Disease[MeSH Terms]) OR (Alzheimer Disease[Title/Abstract])) OR ((((dementia[MeSH Terms]) OR (dementia[Title/Abstract])) OR (mild cognitive impairment[Title/Abstract])) OR (cognit*[Title/Abstract]))) | 918 |
| **Embase** | |
| **Terms** | **Total** |
| ('hearing aid'/exp OR 'hearing aids':ab,ti) AND ('alzheimer disease'/exp OR 'alzheimer disease':ab,ti OR 'dementia'/exp OR dementia:ab,ti OR 'mild cognitive impairment':ab,ti OR cognit*:ab,ti) | 1530 |
| **Cochrane Library** | |
| **Terms** | **Total** |
| #1 (hearing aids):ti,ab,kw (Word variations have been searched) 1049  #2 MeSH descriptor: [Hearing Aids] explode all trees 452  #3 (Alzheimer Disease):ti,ab,kw (Word variations have been searched) 11616  #4 MeSH descriptor: [Alzheimer Disease] explode all trees 3690  #5 (dementia):ti,ab,kw (Word variations have been searched) 14455  #6 MeSH descriptor: [Dementia] explode all trees 6539  #7 (mild cognitive impairment):ti,ab,kw (Word variations have been searched) 4920  #8 (cognit*):ti,ab,kw (Word variations have been searched) 87925  #9 #2 OR #1 1181  #10 #4 OR #3 OR #6 OR #5 OR #7 OR #8 98496  #11 #9 AND #10 200 | 194 |

**Supplementary Table S2. MINORS for risk of bias assessment in non-randomized studies of interventions (NRSIs).**

| Methodological item for non-randomized studies | Tesch-Römer, 1997 | van Hooren et al., 2005 | Doherty and Desjardins, 2015 | Allen et al., 2003 | Acar et al., 2011 | Magalhães and Iório, 2011 | Desjardins, 2016 | Anzivino et al., 2019 | Sarant et al., 2020 | Boi et al., 2012 |
| --- | --- | --- | --- | --- | --- | --- | --- | --- | --- | --- |
| 1. A clear stated aim | 2 | 2 | 2 | 2 | 2 | 2 | 2 | 2 | 2 | 2 |
| 2. Inclusion of consecutive patients | 2 | 2 | 2 | 2 | 2 | 2 | 2 | 2 | 2 | 2 |
| 3. Prospective collection of data | 2 | 2 | 2 | 2 | 2 | 2 | 2 | 2 | 2 | 2 |
| 4. Endpoints appropriate to the aim of the study | 2 | 2 | 2 | 2 | 2 | 2 | 2 | 2 | 2 | 2 |
| 5. Unbiased assessment of the study endpoint | 0 | 0 | 0 | 0 | 0 | 0 | 0 | 0 | 0 | 0 |
| 6. Follow-up period appropriate to the aim of the study | 2 | 2 | 1 | 2 | 1 | 2 | 2 | 2 | 2 | 2 |
| 7. Loss to follow up less than 5% | 1 | 1 | 0 | 1 | 0 | 2 | 2 | 1 | 0 | 1 |
| 8. Prospective calculation of the study size | 0 | 0 | 0 | 0 | 0 | 0 | 0 | 0 | 0 | 0 |
| Additional criteria in the case of comparative studies | | | | | | | | | | |
| 9. An adequate control group | 2 | 2 | 2 | - | - | - | - | - | - | - |
| 10. Contemporary groups | 2 | 2 | 2 | - | - | - | - | - | - | - |
| 11. Baseline equivalence of groups | 2 | 2 | 0 | - | - | - | - | - | - | - |
| 12. Adequate statistical analyses | 2 | 2 | 2 | - | - | - | - | - | - | - |

Table notes: MINORS: Methodological item for non-randomized studies. The items are scored 0 (not reported), 1 (reported but inadequate) or 2 (reported and adequate).
